# Hair Growth and Health Promoting Effects of Standardized *Ageratum conyzoides* Extract in Human Follicle Dermal Papilla Cells and in C57BL/6 Mice

**DOI:** 10.3390/nu17162617

**Published:** 2025-08-12

**Authors:** Jong-Hwan Lim, Chunsik Yi, Eun-Hye Chung, Ji-Soo Jeong, Jin-Hwa Kim, So-Young Boo, Su-Ha Lee, Je-Won Ko, Tae-Won Kim, Young-Hun Kim

**Affiliations:** 1 InnoTNB Co., Ltd., 815, 8, Beobwon-ro 8-gil, Songpa-gu, Seoul 05855, Republic of Korea; ycs321@innotnb.co.kr; 2 BK21 FOUR Program, College of Veterinary Medicine, Chungnam National University, 99 Daehak-ro, Daejeon 34131, Republic of Korea; ksissb1293@gmail.com (E.-H.C.); jisooj9543@gmail.com (J.-S.J.); jinhwa926@g.cnu.ac.kr (J.-H.K.); labong1966@gmail.com (S.-Y.B.); suhai2729@gmail.com (S.-H.L.); rheoda@cnu.ac.kr (J.-W.K.); taewonkim@cnu.ac.kr (T.-W.K.); 3 Department of Animal Nursing Science, Woosong Infomation College, 141, Dongdaejeon-ro, Dong-gu, Daejeon 34606, Republic of Korea; 86vetlover@naver.com

**Keywords:** *Ageratum conyzoides*, hair loss, hair growth, dermal papilla cells, 5α-reductase activity, estrogen receptor signaling, Wnt/β-catenin pathway, reactive oxygen species, antioxidant activity, C57BL/6 mice

## Abstract

**Background/Objectives**: Hair loss, driven by disrupted hair cycles, age-related hormonal imbalances, and oxidative stress, poses significant psychological challenges, necessitating the development of safe and effective therapies. This research investigates the trichogenic potential and underlying mechanisms of a standardized *Ageratum conyzoides* extract (ACE) using human follicle dermal papilla cells (HFDPCs) and C57BL/6 mice as models. **Methods**: HFDPCs were treated with ACE to assess its effects on 5α-reductase activity, estrogen receptor (ERα/ERβ) signaling, and activation of Wnt/β-catenin and MAPK pathways. Reactive oxygen species (ROS) levels and antioxidant enzyme expression were also evaluated. In vivo, C57BL/6 mice were administered ACE orally, and hair regrowth, follicle number and depth, and histological changes were measured. **Results**: In HFDPCs, ACE inhibited 5α-reductase activity, modulated ERα and ERβ signaling, and activated Wnt/β-catenin and MAPK pathways. ACE treatment at 100 μg/mL significantly increased β-catenin, *p*-GSK3β, and vascular endothelial growth factor (VEGF) expression (*p* < 0.01) and decreased Dickkopf-related protein-1 (DKK-)1 expression (*p* < 0.05). It also upregulated VEGF and other hair-growth-related factors and exhibited substantial antioxidant properties by reducing reactive oxygen species (ROS) and elevating the expression of antioxidant enzymes, notably SOD2 at 100 μg/mL. In C57BL/6 mice, oral administration of ACE significantly increased hair regrowth, with the 50 mg/kg group showing the most prominent effects, including increased hair follicle number and depth compared to the negative control group (*p* < 0.05). These effects were observed to be dose-dependent and comparable to those of minoxidil. Histological analysis confirmed enhanced anagen-phase follicle development. **Conclusions**: These findings highlight ACE’s multifaceted biological activity in promoting hair growth through hormonal modulation, pathway activation, and antioxidant protection, positioning it as a promising natural supplement for hair growth and health, although further clinical studies are required to confirm its efficacy in humans.

## 1. Introduction

Hair serves as a critical biomarker of human health and appearance, reflecting physiological and pathological states [1]. Similar to age-related skin changes, such as wrinkling and pigmentation, hair undergoes alterations, including thinning, reduced elasticity, dryness, graying, and loss [2,3,4]. The rising prevalence of age-related hair loss, combined with increased exposure to environmental stressors due to industrial advancements, has heightened the demand for products that promote hair growth and enhance hair health [1,2,3,4].

Hair consists of two primary components: hair follicles (HFs), which regulate hair growth, and the hair shaft, the visible portion of the hair. Hair growth is governed by interactions between HF dermal papilla cells (HFDPCs) and epithelial cells, with HFs cycling through continuous phases of growth (anagen), regression (catagen), and rest (telogen). The anagen phase, which lasts approximately three years in adult scalps, is the dominant stage of the hair cycle [5,6,7]. Hair loss occurs when these cycles are disrupted, often due to premature transitions from anagen to telogen triggered by factors such as androgen hormones [4].

The current pharmacological treatments for hair loss, including finasteride and minoxidil, have limitations [8,9,10,11,12]. Finasteride, a 5-α-reductase inhibitor prescribed for androgenetic alopecia, inhibits testosterone conversion to dihydrotestosterone but is associated with sexual dysfunction in some users [11]. Minoxidil, although widely used despite its partially understood mechanism of action, is linked to adverse effects, such as weight gain, edema, scalp irritation, scaling, respiratory difficulties, and mild tachycardia [12]. These limitations have spurred interest in safe, effective natural alternatives, particularly herbal plant extracts, as potential remedies for hair loss [13].

*Ageratum conyzoides* L., commonly known as Billy Goat Weed, is an annual herb widely distributed across tropical and subtropical regions, including Africa, Asia, and South America [13]. Traditionally used for skin disorders, gastrointestinal issues, headaches, rheumatism, pneumonia, and wound healing, it contains bioactive phytochemicals, such as alkaloids, flavonoids, and terpenes [14,15,16,17,18]. Previous studies in humans suggest that *Ageratum conyzoides* promotes hair growth and reduces hair loss [18]. However, its underlying mechanisms remain incompletely understood.

In this study, we investigated the mechanism of action of standardized ACE by evaluating its effects on the expression of antioxidant and trichogenic regulatory mediators in cultured dermal papilla cells derived from human hair follicles. Additionally, its impact on hair growth and hair composition was assessed in a C57BL/6 mouse model.

## 2. Materials and Methods

### 2.1. Preparation of Standardized Ageratum conyzoides Extract and Phytochemical Analysis

Standardized *Ageratum conyzoides* extract was provided by Gencor Pacific (Hongkong, China). The aerial parts of *Ageratum conyzoides* (India) were extracted twice with ethanol:water (90:10, *v*/*v*) at 75–80 °C for 4 h. The filtrate was concentrated under vacuum at 80–90 °C, spray-dried, and blended with 5% maltodextrin.

### 2.2. Cell Culture

HFDPCs and human hair outer root sheath cells (HHORSCs) obtained from PromoCell (Heidelberg, Germany) were cultured in Keratinocyte Growth Medium 2 (PromoCell, Heidelberg, Germany) supplemented with SupplementMix (PromoCell, Heidelberg, Germany) and 1% penicillin/streptomycin (Thermo Fisher Scientific, Waltham, MA, USA). Cells were maintained at 37 °C in a humidified incubator with 5% CO_2_.

### 2.3. Cell Viability Assays

HFDPCs were seeded at 2 × 10^4^ cells/mL in 96-well plates and treated with 0.1% DMSO or ACE (12.5–400 µg/mL) for 72 h. MTT solution (5 mg/mL) was added, incubated for 3 h, and formazan was dissolved in DMSO. Absorbance was measured at 570 nm using a microplate reader (Tecan, Männedorf, Switzerland).

### 2.4. Quantitative Real-Time PCR (qPCR)

HFDPCs treated with ACE (25–100 µg/mL) for 24 h were lysed, and RNA was isolated with TRIzol (Invitrogen, Carlsbad, CA, USA), and 1 µg of RNA was subsequently reverse-transcribed into cDNA (RevertAid Kit, Thermo Fisher Scientific). qPCR was performed using TaqMan Universal PCR Master Mix (Applied Biosystems, Foster City, CA, USA) on a StepOnePlus system with TaqMan Gene Expression Assays with Taqman primers according to the cell type, WNT3 (Hs00902257_m1), IGF-1 (Hs01547620_m1), VEGF (Hs00900055_m1), KGF/FGF7 (Hs00940265_m1), and Glyceraldehyde-3-phosphate dehydrogenase (GAPDH (Hs02786624_g1). Expression was calculated via the 2^−ΔΔCt^ method, normalized to GAPDH.

### 2.5. Immunoblotting

HFDPCs (2 × 10^5^ cells/well, 6-well plates) were treated with ACE (25–100 µg/mL) or finasteride (10 µM) for 24 h. Lysates were prepared using tissue lysis reagent (Sigma-Aldrich, Saint Louis, MO, USA), and protein was quantified using Bradford reagent (Bio-Rad, Hercules, CA, USA). Proteins (10% SDS-PAGE) were transferred to PVDF membranes (Millipore, Burlington, MA, USA), blocked with 5% non-fat milk in TBST, and probed with antibodies against 5α-reductase 2, ERα, ERβ, *p*-p38, p38, *p*-ERK, ERK, SOD1, SOD2, GPx, Keratin-1, Keratin-2, and β-actin (Cell Signaling Technology, Danvers, MA, USA; 1:1000) at 4 °C overnight, and subsequently, secondary antibodies were applied at a 1:10,000 dilution for 1 h at room temperature (approximately 23 °C). Bands were visualized with a LuminoGraph (Atto, Osaka, Japan) and quantified using ImageJ, https://imagej.net/ij/index.html (accessed on 10 August 2025) (NIH, Bethesda, MD, USA), normalized to β-actin.

### 2.6. ALDH2 Activity Assay

HHORSCs (2 × 10^5^ cells/well, 6-well plates) treated with ACE (25–100 µg/mL) for 24 h were analyzed for ALDH2 activity using the ALDEFLUOR™ Kit (STEMCELL Technologies, Vancouver, BC, Canada) per the manufacturer’s instructions.

### 2.7. Animal Experiments

Six-week-old male C57BL/6 mice (18–20 g; Orientbio, Seongnam, Republic of Korea) were acclimatized for 7 days under standard conditions (22 ± 3 °C, 50 ± 5% humidity, 12 h light/dark cycle). After removing the dorsal hair to synchronize the hair growth cycle, mice were randomly assigned to groups: distilled water (NC, oral administration), 3% minoxidil (PC, topical treatment), or ACE (10, 20, 50 mg/kg, oral administration) for 21 days. A total of 15 C57BL/6 mice (*n* = 3 per group) were used in this study. Dorsal skin was photographed on days 1, 7, 14, and 21. Skin samples were fixed in 4% paraformaldehyde. Experiments followed Chungnam National University IACUC guidelines (Approval no. 202403A-CNU-038). The degree of hair regrowth was evaluated using a 4-point scale (0–3) based on changes in dorsal skin coloration. A score of 0 denoted no regrowth (pink skin); 1 corresponded to grayish skin (early stage regrowth); 2 represented a darker gray tone (intermediate-to-advanced regrowth); and 3 signified black skin, indicating complete hair coverage. The hair regrowth score was calculated by measuring the ratio of the hair regrowth area to the total area, and the sum of the area ratio multiplied by the score was averaged for each group [19]. The protein expression of keratin type 1 and type 2 in dorsal skin samples from BALB/c mice was analyzed using Western blot.

### 2.8. Histological Analysis

Dorsal skin tissues were collected and fixed in 4% formalin, embedded in paraffin, sectioned at 4 μm, and stained with hematoxylin and eosin (H&E). The number and diameter of hair follicles were quantified in at least three fields per mouse at 100× magnification, and skin thickness was also measured in at least three fields per mouse at 40× magnification using ImageJ image analysis tool.

### 2.9. Statistical Analysis

Data are expressed as mean ± standard deviation. Groups were compared using Student’s *t*-test and one-way analysis of variance, as applicable. All statistical analyses were carried out using Origin version 7.0 software (Microcal Software, Northampton, MA, USA). A *p*-value < 0.05 was considered statistically significant.

## 3. Results

### 3.1. Cell Viability of ACE in Human Follicle Dermal Papilla Cells

To evaluate the cytotoxicity of ACE on DPCs, a cell viability assay was performed across a range of concentrations (0–200 µg/mL). ACE demonstrated no significant cytotoxicity at concentrations up to 100 µg/mL, as cell viability remained comparable to the control group.

### 3.2. Inhibition of 5α-Reductase 2 and Modulation of Estrogen Receptors by ACE in Human Follicle Dermal Papilla Cells

**Figure 1:**
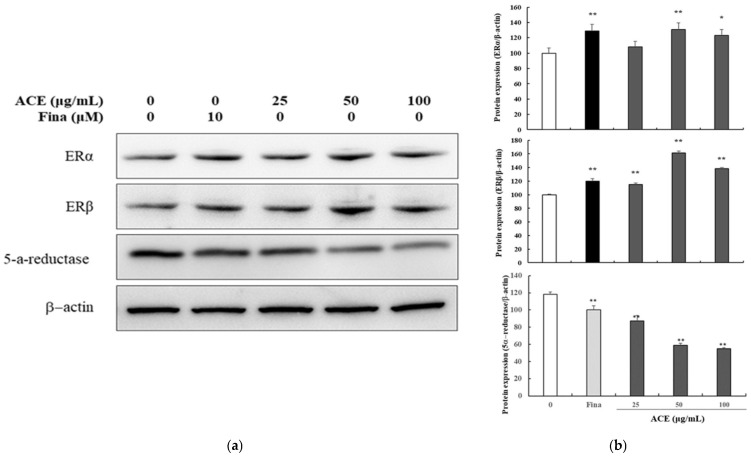
Effects of standardized *Ageratum conyzoides* extract (ACE) on 5α-reductase and estrogen receptor expression in human follicle dermal papilla cells (HFDPCs). (**a**) Representative Western blot images showing protein expression levels of 5α-reductase, ERα, and ERβ in HFDPCs after treatment with ACE (25, 50, or 100 μg/mL) or finasteride (10 μM) for 24 h. Protein levels were normalized to β-actin; (**b**) Band intensities were quantified using ImageJ software. Abbreviations: NC, DMSO-treated control; ACE, standardized *Ageratum conyzoides* extract; Fina, finasteride. Values represent mean ± SD of three independent replicates. Statistical significance: * *p* < 0.05, ** *p* < 0.01 vs. NC group.

The effect of ACE on 5α-reductase, a key enzyme implicated in androgenetic alopecia, was evaluated in HPDPCs. Western blot analysis revealed a dose-dependent reduction in 5α-reductase protein levels compared to the untreated control (Figure 1), suggesting a potential mechanism for its anti-alopecia effects. Furthermore, a significant, dose-dependent upregulation of ERβ expression was shown in the ACE-treated cells. In contrast, ERα expression, although lower than ERβ, exhibited a statistically significant increase compared to the untreated control.

### 3.3. Modulation of Hair Growth Pathways by ACE in HFDPCs

**Figure 2:**
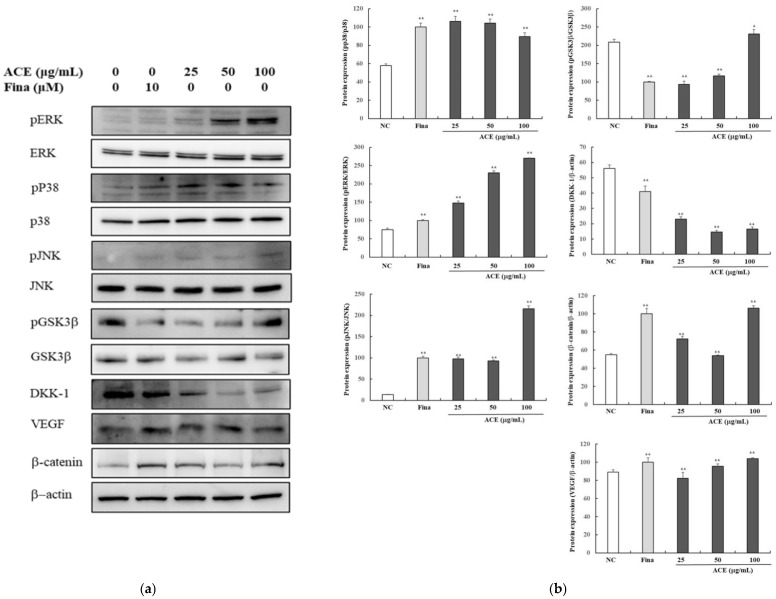
Multi-target hair growth effects of standardized *Ageratum conyzoides* extract (ACE) via MAPK and Wnt/β-catenin signaling in human follicle dermal papilla cells (HFDPCs). (**a**) Representative Western blot images showing protein expression levels of phosphorylated p38, ERK, JNK, and β-catenin in HFDPCs after treatment with ACE (25, 50, or 100 μg/mL) or finasteride (10 μM) for 24 h. Protein levels were normalized to β-actin; (**b**) Band intensities were quantified using ImageJ software. Abbreviations: NC, DMSO-treated control; ACE, standardized *Ageratum conyzoides* extract; Fina, finasteride. Values represent mean ± SD of three independent replicates. Statistical significance: * *p* < 0.05, ** *p* < 0.01 vs. NC group.

**Figure 3:**
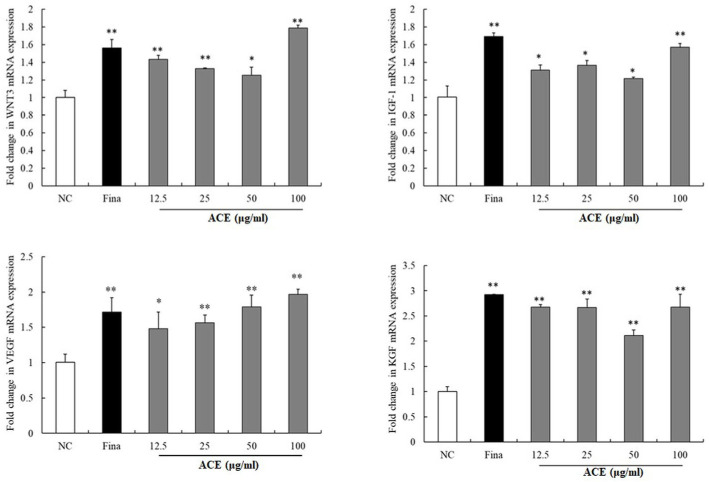
Effects of standardized *Ageratum conyzoides* extract (ACE) on Wnt/β-catenin signaling and hair growth factor expression in human follicle dermal papilla cells (HFDPCs). HFDPCs were treated with ACE (12.5, 25, 50, or 100 μg/mL) or finasteride (10 μM) for 24 h. mRNA expression of WNT3 and hair growth factors was quantified by qPCR using specific primers, normalized to GAPDH via the 2^−ΔΔCt^ method. Abbreviations: NC, DMSO-treated control; ACE, standardized *Ageratum conyzoides* extract; Fina, finasteride. Values represent mean ± SD of three independent replicates. Statistical significance: * *p* < 0.05, ** *p* < 0.01 vs. NC group.

To investigate the mechanisms of ACE in promoting hair growth, MAPK pathway components (*p*-p38/p38, *p*-ERK/ERK, *p*-JNK/JNK), Wnt/β-catenin signaling (β-catenin, *p*-GSK3β, DKK-1), and VEGF were assessed by Western blot (Figure 2), and mRNA levels of WNT3, IGF-1, and KGF were evaluated by qPCR (Figure 3). Western blot analysis showed dose-dependent increases in β-catenin, *p*-GSK3β, and VEGF with ACE (*p* < 0.01; Figure 2), comparable to finasteride. A significant reduction in DKK-1 expression was observed (*p* < 0.05; Figure 2). Total GSK3β levels remained unchanged at 100 µg/mL. Upon treatment with 100 µg/mL, a notable elevation in the phosphorylation ratios of ERK and p38 was observed within the MAPK signaling cascade (*p* < 0.01; Figure 2), whereas the phosphorylation status of JNK remained statistically unchanged. qPCR analysis demonstrated upregulated expression of WNT3, IGF-1, VEGF, and KGF mRNA levels in ACE-treated HFDPCs. mRNA expression of WNT3, IGF-1, and KGF was markedly upregulated at 100 µg/mL ACE, while VEGF showed a significant, dose-dependent increase in the ACE-treated group compared to controls (*p* < 0.05; Figure 3), with effects comparable to those of finasteride.

### 3.4. Enhancement of Antioxidant Enzyme Expression by ACEs

**Figure 4:**
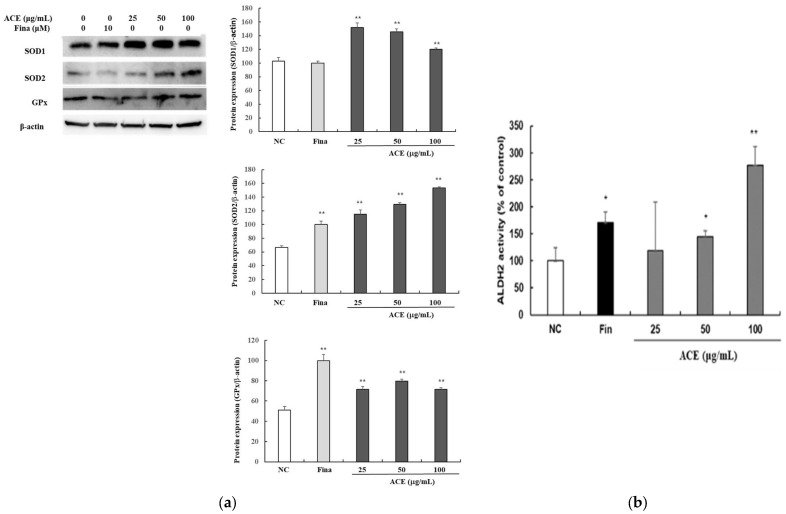
Antioxidant and detoxification effects of standardized *Ageratum conyzoides* extract (ACE) in human follicle dermal papilla cells (HFDPCs) and human hair outer root sheath cells (HORSCs). (**a**) Representative Western blot images showing protein expression levels of SOD1, SOD2, and GPx in HFDPCs after treatment with ACE (25, 50, or 100 μg/mL) or finasteride (10 μM) for 24 h. Protein levels were normalized to β-actin, and band intensities were quantified using ImageJ software; (**b**) HORSCs were treated similarly for 24 h, and ALDH2 activity was measured using the ALDEFLUOR™ Kit (STEMCELL Technologies, Canada). Band intensities were quantified using ImageJ software. Abbreviations: NC, DMSO-treated control; ACE, standardized *Ageratum conyzoides* extract; Fina, finasteride. Values represent mean ± SD of three independent replicates. Statistical significance: * *p* < 0.05, ** *p* < 0.01 vs. NC group.

To assess the antioxidant potential of ACE, the expression of SOD1, SOD2, and GPx was evaluated in HFDPCs via Western blot (Figure 4). ACE increased their protein levels, most notably SOD2 at 100 µg/mL, compared to controls (Figure 4a). In addition, the effect of ACE on ALDH2 activity, a marker of cellular detoxification and stress response, was assessed in HORSCs (Figure 4b). A 60 min ALDH2 assay showed dose-dependent activity increases with ACE treatment.

### 3.5. Hair Growth Promotion by ACE in C57BL/6 Mice

**Figure 5:**
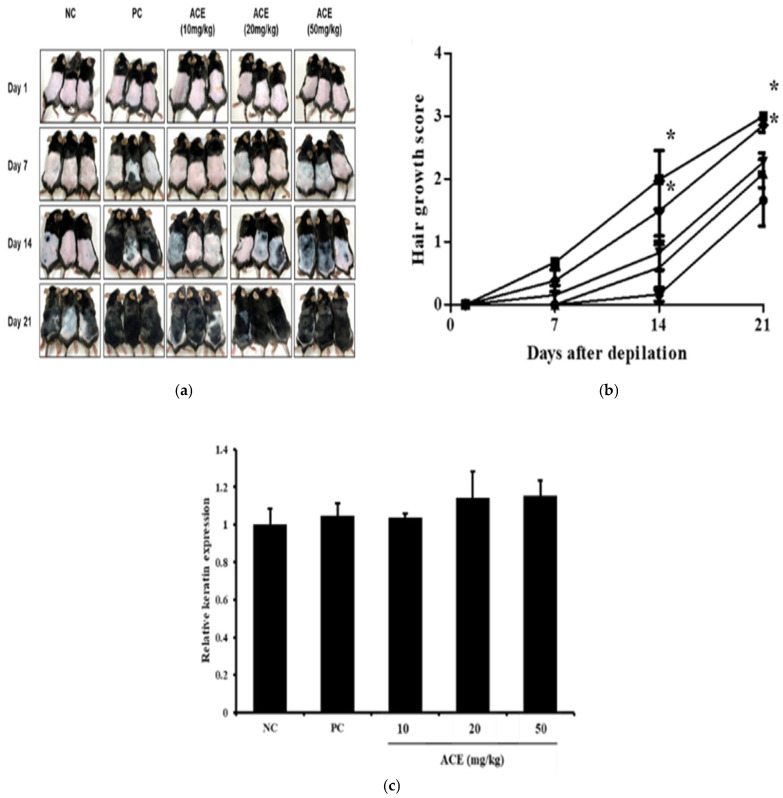
Hair-growth-promoting effects of standardized *Ageratum conyzoides* extract (ACE) in C57BL/6 mice. (**a**) Representative images of dorsal hair regrowth in C57BL/6 mice after 0, 7, 14, and 21 days of treatment. The negative control (NC) group received distilled water (vehicle) orally; the positive control (PC) group was treated topically with minoxidil; and test groups were administered ACE orally at 10, 20, or 50 mg/kg/day; (**b**) Hair regrowth scores were calculated as the ratio of regrowth area to total shaved area, weighted by score, and averaged for NC (●), PC (■), ACE 10 mg/kg (▲), ACE 20 mg/kg (▼), and ACE 50 mg/kg (◆) groups; (**c**) Protein expression of keratin type 1 and type 2 in dorsal skin samples from C57BL/6 mice was analyzed using Western blot. Relative expression levels were normalized to β-actin and then to the NC group. Data are presented as mean ± SD. Statistical significance is indicated: * *p* < 0.05.

To evaluate the hair-growth-promoting effects of standardized ACE, C57BL/6 mice were orally administered ACE at doses of 10, 20, and 50 mg/kg for 21 days. The hair-promoting activity of ACE was assessed using the C57BL/6 murine model. The NC group exhibited minimal hair regrowth, with sparse and uneven hair coverage by day 21 (Figure 5). In contrast, the PC group displayed robust hair regrowth, characterized by dense and uniform hair coverage across the shaved area. ACE treatment induced a dose-dependent improvement in hair regrowth. ACE treatment at a dose of 50 mg/kg elicited the most prominent effect among the ACE-treated groups, inducing hair regrowth with a rapid rate and uniformity comparable to that of the positive control (Figure 5a,b). Keratin expression in dorsal skin was measured to assess follicular activity at the molecular level after 21 days of treatment (Figure 5c). The NC group exhibited low keratin levels, consistent with hair follicles in the telogen phase, while ACE-treated groups showed increased keratin expression in a dose-dependent manner, indicative of active anagen-phase hair growth and enhanced hair shaft formation.

**Figure 6:**
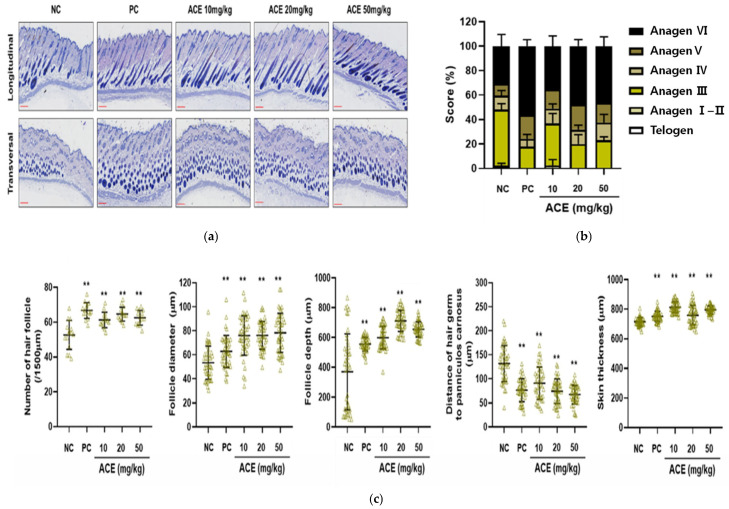
Histological analysis of dorsal skin in C57BL/6 mice. (**a**) Representative hematoxylin and eosin (H&E)-stained longitudinal and transversal sections of dorsal skin. Scale bar = 300 μm; (**b**) Anagen scores; (**c**) Hair follicle counts, hair follicle diameters, hair follicle depth, distance of hair germ to panniculus carnosus, and skin thickness were quantified from at least three fields per sample. The negative control (NC) group received oral distilled water (vehicle); the positive control (PC) group was treated topically with minoxidil; and test groups were orally administered *Ageratum conyzoides* extract (ACE) at 10, 20, or 50 mg/kg/day. Data are presented as mean ± SD. Statistical significance is indicated: ** *p* < 0.01 vs. NC group.

Histological analysis demonstrated that ACE treatment significantly increased hair follicle number and size, with deeper follicular penetration into the dermis, indicating progression into the anagen phase (Figure 6a,b). In the NC group, most hair follicles remained in the telogen phase. In contrast, ACE-treated groups displayed a clear shift toward the anagen phase, accompanied by follicle enlargement and elongation, particularly in the 20 and 50 mg/kg groups (Figure 6a,b). ACE-treated groups showed a dose-dependent increase in follicle number, diameter, depth, and skin thickness relative to the untreated controls (*p* < 0.05; Figure 6c).

## 4. Discussion

Hair loss, including androgenetic alopecia (AGA) and female pattern hair loss (FPHL), is commonly observed and results from the interplay of hereditary, hormonal, and environmental influences [4]. It is characterized by androgen-mediated miniaturization of scalp hair follicles, leading to progressive hair thinning in distinct patterns [4]. These conditions are associated with significant psychosocial impacts, including reduced self-esteem and social withdrawal, fueling demand for effective treatments [1,4]. The current FDA-approved therapies, oral finasteride and topical minoxidil, target specific mechanisms: finasteride inhibits 5α-reductase to reduce dihydrotestosterone (DHT) levels, while minoxidil enhances perifollicular blood flow [8,9]. However, these treatments have limitations. Finasteride does not address the inflammatory or oxidative stress responses contributing to follicular regression, and minoxidil lacks direct effects on intracellular signaling or inflammatory cytokine activity [10,11,12]. Consequently, there is growing interest in natural extracts, such as standardized ACE, which may offer multi-targeted therapeutic benefits for hair growth and follicle health.

*Ageratum conyzoides* is a medicinal plant traditionally recognized for its anti-inflammatory and analgesic properties, with proven efficacy in suppressing prostaglandins and inflammatory cytokines [14,15]. Previous studies have suggested its potential to inhibit androgen signaling and 5α-reductase activity in testosterone-induced benign prostatic hyperplasia models [20]. Consistent with prior research, ACE showed dual activity by inhibiting 5α-reductase and modulating estrogen receptor signaling, targeting multiple etiological pathways of hair loss. This multi-target approach distinguishes ACE from conventional therapies, offering the potential to mitigate both androgenic and inflammatory factors in hair loss.

The Wnt/β-catenin signaling pathway plays a pivotal role in hair follicle development and growth [21,22,23,24]. β-catenin, expressed in both mesenchymal and epithelial cells of the hair follicle, is essential for sustaining follicle development and promoting the proliferation and differentiation of hair follicle stem cells [22,23,24,25,26]. Additionally, GSK-3β signaling regulates various biological processes, including cell proliferation, hair growth, and regeneration [27,28]. IGF-1 activates Wnt/β-catenin signaling, which in turn upregulates VEGF, a key hair growth factor [23,24,25]. Notably, IGF-1 itself is a critical regulator of hair cycle transitions and differentiation [25]. Many studies have found many natural compounds, including flavonoid, chalcone, terpenoids, which activate the Wnt/β-catenin pathway or its crosstalk, which may be potential therapies for treating hair loss [23]. *Ageratum conyzoides* was reported to contain various phytochemicals, including alkaloids, flavonoids, tannins, and terpenoids, which are known to exhibit a wide range of pharmacological activities [14,16]. In the current study, ACE activated the Wnt/β-catenin pathway, a key regulator of the hair follicle cycle and regeneration. In the absence of Wnt signaling, β-catenin is degraded by the GSK-3β/APC/Axin complex. However, when Wnt is activated, stabilized β-catenin translocates to the nucleus, inducing the expression of genes such as cyclin D1 and promoting cell proliferation. Additionally, ACE enhanced MAPK signaling, supporting the proliferation, polarity, and migration of dermal papilla cells. ACE also upregulated growth factors such as VEGF, IGF-1, and KGF, contributing to angiogenesis and hair follicle development. These molecular mechanisms suggest that ACE may promote hair growth through coordinated signaling pathways.

Hair loss is exacerbated by oxidative stress from ROS, induced by environmental factors such as ultraviolet radiation, smoking, and pollution, as well as stress-mediated prolongation of the telogen phase [2,3]. ACE significantly upregulated antioxidant enzymes, including superoxide dismutase (SOD1, SOD2) and GPx, strengthening the follicular antioxidant defense system. Furthermore, ACE enhanced ALDH2-mediated detoxification in HORSCs, suggesting a protective role against stress-induced follicular damage.

The C57BL/6 mouse is widely used as an animal model for evaluating hair health and hair growth effects [29,30]. In the present study, ACE promoted hair regrowth in C57BL/6 mice by facilitating the telogen-to-anagen transition and potentially extending the anagen phase, consistent with its multi-targeted effects observed in HFDPCs. These findings underscore ACE’s dose-dependent enhancement of hair regeneration through hormonal modulation, pathway activation, and antioxidant protection. However, several limitations should be considered. Although C57BL/6 mice and HFDPCs offer useful preclinical models, they do not fully replicate the complex structural, hormonal, and immunological environment of the human scalp. In addition, the oral doses administered in mice (e.g., 10–50 mg/kg) may not directly correspond to feasible or effective doses in humans; thus, clinical trials are required to confirm ACE’s efficacy and safety as a natural supplement for hair growth and health.

## 5. Conclusions

ACE represents a promising therapeutic candidate for hair loss, leveraging its ability to modulate androgen and estrogen signaling, activate Wnt/β-catenin and MAPK pathways, and enhance antioxidant defenses. These multifaceted effects position ACE as a potential alternative or complementary approach to existing treatments, pending further clinical evaluation.

## Data Availability

Data are not publicly available due to privacy concerns.

## Abbreviations

ACE	*Ageratum conyzoides* extract
HFDPCs	Human follicle dermal papilla cells
ER	Estrogen receptor
ROS	Reactive oxygen species
VEGF	Vascular endothelial growth factor
DKK-1	Dickkopf-related protein-1
SOD	Superoxide dismutase
HFs	Hair follicles
HHORSCs	Human hair outer root sheath cells
GAPDH	Glyceraldehyde-3-phosphate dehydrogenase
H&E	Hematoxylin and eosin
AGA	Androgenetic alopecia
FPHL	Female pattern hair loss
DHT	Dihydrotestosterone
